# β-amyloid and β-amyloid precursor protein synthesis and processing: effects of exercise and exercise-related injury

**DOI:** 10.3389/fncel.2026.1735542

**Published:** 2026-02-09

**Authors:** Weizhe Zhen, Yanfeng Xing, Yongqin Li, Junhang Hao, Linglei Kong, Hongjun Zhen

**Affiliations:** 1 Graduate School, Capital Medical University, Beijing, China; 2 Department of Orthopedics, Handan Chinese Medicine Hospital, Handan, Hebei, China

**Keywords:** exercise, exercise-related injury, processing, synthesis, β-amyloid, β-amyloid precursor protein

## Abstract

During the processing and assembly of β-amyloid precursor protein (APP), the amyloidogenic pathway represents a crucial component of Alzheimer’s disease (AD) pathogenesis. The non-amyloidogenic pathway does not generate toxic Aβ. For a long time, research has delved deeply into both pathways, elucidating numerous important details and mechanisms within these processes. Scientists and clinicians have sought to design effective therapeutic interventions based on these mechanisms. However, this endeavor has encountered numerous setbacks, resulting in no currently available drugs capable of reversing AD progression. Regarding APP processing and assembly, we are curious whether daily activities influence these processes. We focus on exercise as a daily activity, systematically exploring whether it affects APP processing and assembly and the underlying mechanisms. Furthermore, we examine alterations in APP processing and assembly in exercise-related injury disorders, summarizing and analyzing existing research. We discuss promising future research directions, aiming to contribute to preventing adverse outcomes following exercise-related injuries.

## Introduction

Extracellular neuroinflammatory plaques composed of β-amyloid (Aβ) are one of the primary pathological manifestations of Alzheimer’s disease(AD) (Zheng and Wang, 2025). Although currently available treatments for AD are limited, some therapeutic approaches aim to exert their effects by clearing Aβ plaques. As basic research continues to advance, Aβ has increasingly been found to be closely associated with numerous other diseases and pathological changes (Stein et al., 2015; Jang et al., 2022). This has also driven research to focus on the formation and aggregation processes of Aβ plaques. Aβ is generated through the proteolytic processing of the β-amyloid precursor protein (APP). Abnormalities in this process are widely recognized as one of the primary causes of AD.

Exercise is an indispensable part of human life. Many people maintain regular exercise habits, and even those without structured exercise plans still achieve a certain amount of physical activity through commuting, shopping, and other daily routines. Whether exercise influences the APP assembly process remains inconclusive, as current research lacks sufficient evidence to reach a universally accepted conclusion. Nevertheless, this represents a highly significant avenue for further investigation. Gaining deeper insights into how different types of exercise, varying in frequency, duration, and intensity, affect the APP assembly process over sustained periods could profoundly and positively impact our ability to provide daily exercise recommendations for individuals with Aβ-related diseases. It may even pave the way for developing novel physical therapies.

Exercise can be categorized into aerobic and anaerobic activities. For anaerobic exercise, the rapid bursts of short-term strength exertion make beginners or those without proper warm-ups highly susceptible to sports injuries. Even with aerobic exercise, prolonged strenuous activity and improper muscle relaxation techniques can lead to joint and muscle strain, or even skeletal damage, among participants. While we aim to prevent sports injuries, they remain an inherent and frequent occurrence in athletic activities. During the injury process and subsequent disease progression, whether APP undergoes abnormal hydrolysis, assembly, or modification, and whether Aβ plaques form and aggregate abnormally, remains unknown—yet constitutes a highly intriguing research question.

## β-amyloid precursor protein and its assembly process

β-amyloid precursor protein is a type I transmembrane protein that forms the mammalian APP family alongside APP-like proteins amyloid beta precursor like protein 1 (APLP1) and proteins amyloid beta precursor like protein 2 (APLP2). Biological functions involving APP family members include nervous system development, formation and function of the neuromuscular junction, synaptogenesis, dendritic complexity and spine density, axonal growth and guidance, and synaptic function—including synaptic plasticity, learning, and memory (Müller et al., 2017). APP primarily follows two metabolic pathways: the non-amyloidogenic pathway and the amyloidogenic pathway. The amyloidogenic pathway produces β-Amyloid (Aβ), whose resulting plaques constitute one of the hallmark pathological features of AD. Which pathway APP follows for metabolism depends on the enzyme involved in the first cleavage step. When α-secretase is involved in the first cleavage, the integrity of the Aβ peptide segment is disrupted, thereby preventing the generation of full-length Aβ (Lichtenthaler and Haass, 2004). When β-secretase participates in the first cleavage step, the process of Aβ generation is initiated (Vassar et al., 1999). β-secretase is a key enzyme regulating APP metabolism, working in concert with γ-secretase which performs the second cleavage step to generate Aβ fragments. Its abnormal activation leads to an imbalance in the Aβ42/Aβ40 ratio (Citron, 2002). Gamma-secretase is a multimeric aspartic protease that cleaves the transmembrane region of the β-carboxy-terminal fragment (βCTF) derived from APP, ultimately yielding a series of Aβ peptides of varying lengths (Tomita, 2008; Yonemura et al., 2011; Funamoto et al., 2020). Research on APP has largely been concentrated over the past two decades, with its physiological functions, specificity, and transmembrane domains continually being elucidated (Ashall and Goate, 1994; Tischer and Cordell, 1996). In recent years, research on APP has increasingly focused on them as a breakthrough in clinical therapies. Although no proven effective treatments have been approved yet, multiple novel drugs are being developed and entering clinical trials during the research phase (Koriyama et al., 2021). As novel research techniques are developed, they are also employed as characteristic markers in the construction of cellular models (Tunesi et al., 2020).

## The role and mechanism of APP in the course of AD

β-amyloid precursor protein plays a significant role throughout the entire disease progression of AD and can even be considered one of the key initiators (Chen et al., 2024). Its primary function lies in serving as the source within the “amyloid cascade hypothesis.” Factors such as polygenic mutations alter the process by which APP generates Aβ, leading to increased production of Aβ42, which in turn promotes Aβ deposition (Hardy and Higgins, 1992). However, this does not mark the end of APP’s role in the AD process. As research deepens, Aβ-forming plaques are no longer universally recognized as the primary mechanism of AD pathogenesis, and other mechanisms are gradually gaining acceptance. Among these, Aβ oligomers have emerged as a focal point of intense attention. APP metabolism can generate Aβ oligomers, which not only inhibit long-term potentiation (LTP) and enhance long-term depression (LTD), but can also cause damage to synaptic structures (Selkoe, 2008). Beyond their effects on synapses, Aβ oligomers can also exert neurotoxicity and trigger multifaceted destructive effects such as neuroinflammation (Heneka et al., 2015; Sui et al., 2015). These effects initiated by the APP interact with one another, collectively driving the progression of AD.

## The modulating effect of exercise on the course of Alzheimer’s disease

Physical activity is integral to daily life. Even when we don’t set aside dedicated time for exercise, routine commutes and daily activities often provide a certain level of movement without us realizing it. A growing body of research indicates that exercise can effectively slow cognitive decline in elderly patients with mild to moderate AD (Yu et al., 2021), demonstrating significant potential as a preventive and even therapeutic strategy. There are numerous classification criteria for exercise, such as categorizing it into aerobic and anaerobic activities. Previous studies have focused on whether both aerobic and anaerobic exercise exert beneficial effects on cognitive function and pathological manifestations in AD patients (Table 1). Aerobic exercise is defined as sustained rhythmic activity involving large muscle groups, where oxygen demand does not exceed oxygen supply. Anaerobic exercise encompasses brief, intense activities such as weightlifting, sprinting, and high-intensity interval training, where oxygen demand exceeds oxygen supply (Huang et al., 2023). Resistance training also largely falls under the category of anaerobic exercise (Smith et al., 2019). Based on the research findings, both types of exercise can exert beneficial effects (Alkadhi and Dao, 2018; Liu et al., 2020; Rossi Daré et al., 2020; Gaitán et al., 2021; Zhou et al., 2022; Azevedo et al., 2023; Cui et al., 2023; Sepúlveda-Lara et al., 2024; Liang et al., 2025). In contrast, aerobic exercise has garnered greater research attention, with several randomized controlled trials designed and completed to investigate its effects on AD (Morris et al., 2017). Similarly, exercise duration and intensity are variables of significant interest to researchers. While high-intensity and moderate-intensity exercise do not alter the expression of AD-related genes in cognitively unimpaired older adults, moderate-to-high-intensity exercise effectively alleviates neuropsychiatric symptoms in patients with mild AD (Hoffmann et al., 2016; Marston et al., 2024).

**TABLE 1:** The effects of different types of exercise and injury conditions on β-amyloid precursor protein (APP) processing, β-amyloid (Aβ) levels and cognitive outcomes.

Exercise and related diseases	Classification	APP processing	Aβ level	Cognitive outcomes
Exercise	Aerobic exercise	Decrease	Decrease	Improve
	Anaerobic exercise	Decrease (indirect)	Decrease	Improve
Exercise injury related diseases	Fracture	Unclear	Increase	Impair
	Osteoporosis	Unclear	Increase	Impair
	Muscle injury	Unclear	Unclear	Impair
	Lumbar disk herniation	Unclear	Unclear	May impair

## The impact of exercise on the processing and assembly process of the APP

**FIGURE 1:**
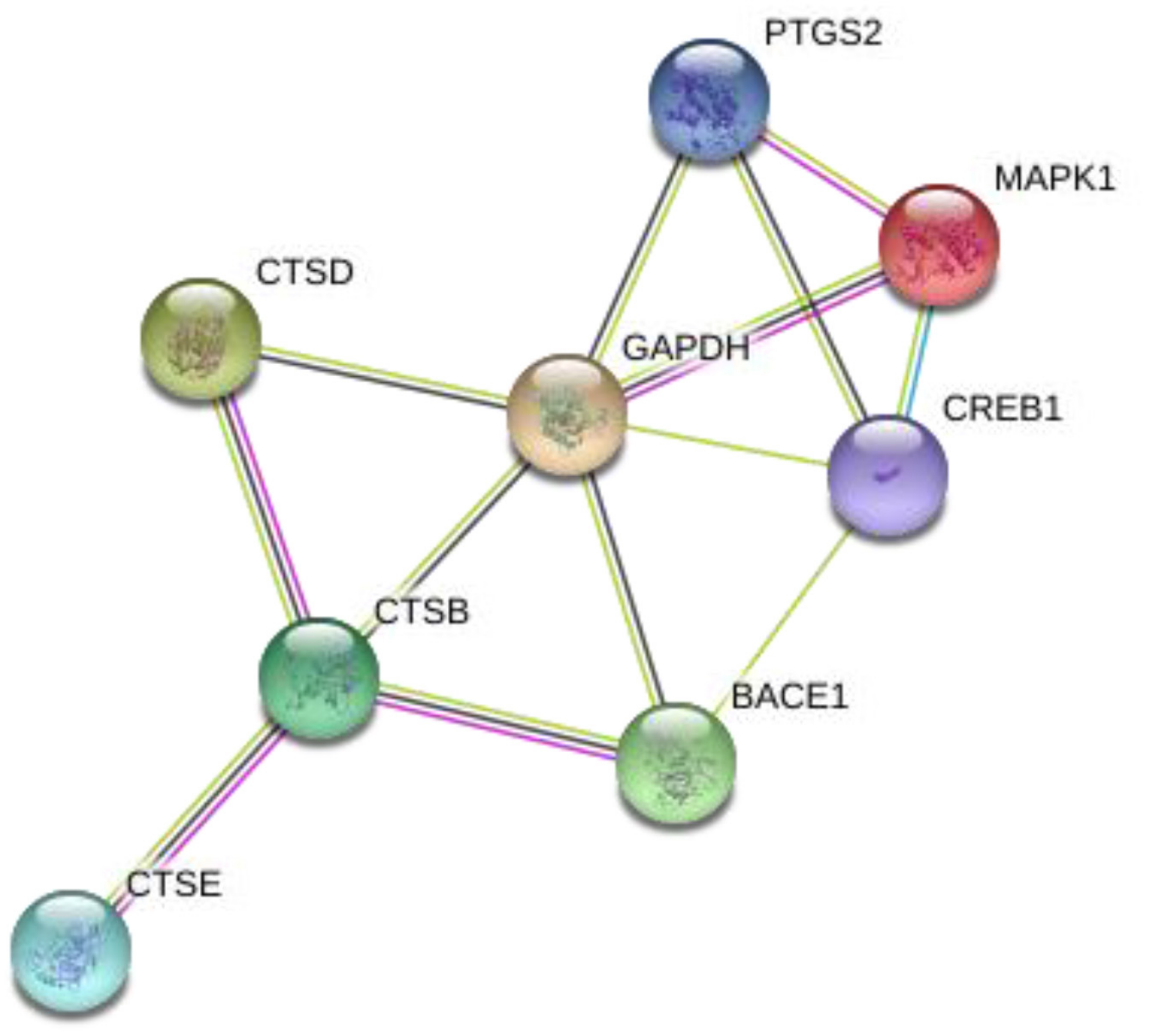
The interaction between key biomolecules. PTGS2, prostaglandin-endoperoxide synthase 2, also known as cyclooxygenase 2 (COX2); MAPK1, mitogen-activated protein kinase 1, also known as extracellular signal-regulated kinase (ERK); CREB1, CAMP response element-binding protein 1; GAPDH, glyceraldehyde-3-phosphate dehydrogenase; CTSB, cathepsins B; CTSD, cathepsins D; CTSE, cathepsins E; BACE1, APP-cleaving enzyme 1. *Draw by String: https://cn.string-db.org/.

**FIGURE 2:**
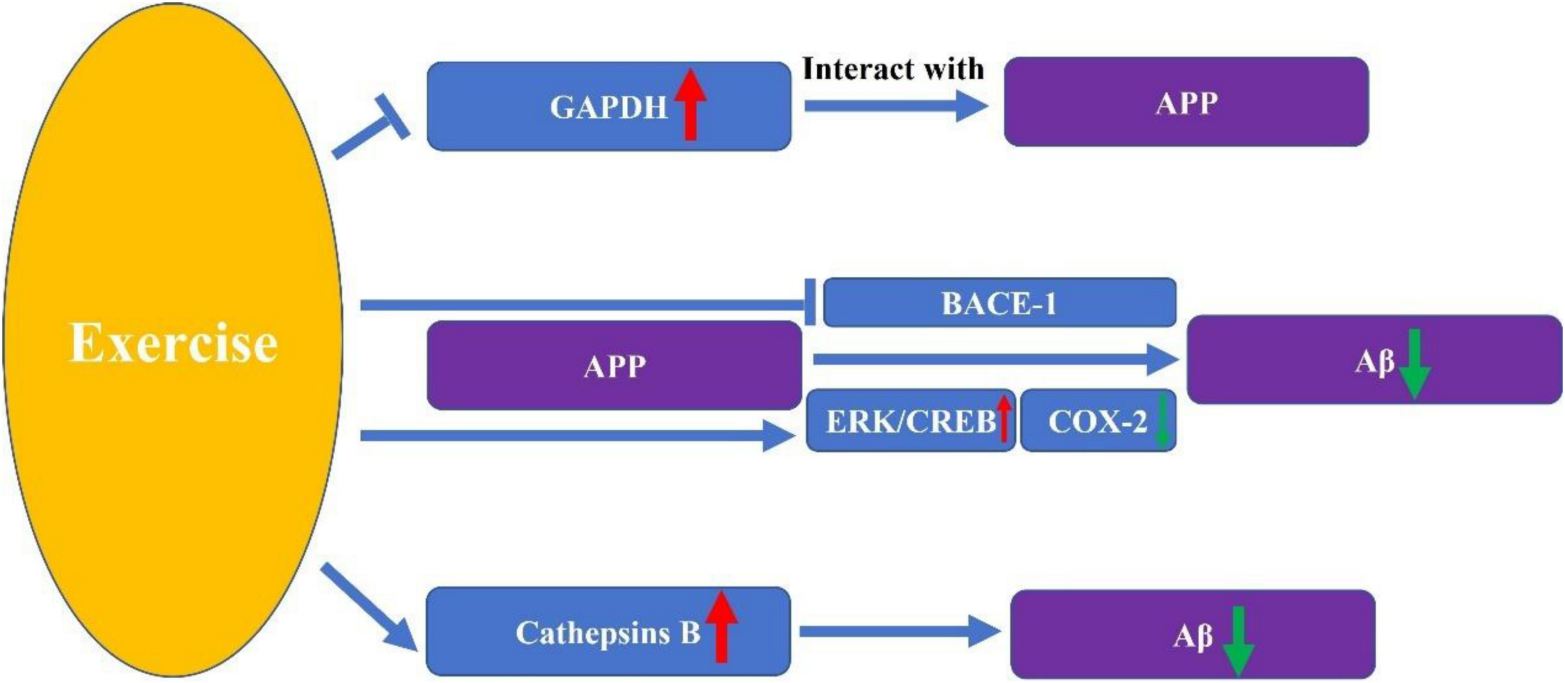
The Key molecular pathways through which exercise influences β-amyloid precursor protein (APP) processing.

The impact of exercise on APP processing remains poorly understood, with insufficient research evidence elucidating the complete mechanisms underlying this process. This leaves significant uncharted territory for future exploration. Current studies directly revealing the mechanisms by which exercise influences APP processing suggest that exercise can downregulate β-site amyloid precursor protein-cleaving enzyme 1 mRNA (Yu et al., 2013). Although studies directly revealing the effects of exercise on APP processing changes are scarce, we can identify several potential mechanisms from other related research that warrant further clarification. These mechanisms often feature key biomolecules as their core components (Figures 1, 2).

### COX-2

Cyclooxygenase (COX) plays a key role in the synthesis of prostaglandin E2 (PGE2) from arachidonic acid (Zhu et al., 2025). COX has three isoforms: COX-1, COX-2, and COX-3. COX-2 is a potent enzyme that, upon stimulation by various inflammatory factors such as cytokines and bacteria, can trigger inflammation and promote prostaglandin synthesis (Yang et al., 2020). Its elevated levels are closely associated with the onset of AD (Moussa and Dayoub, 2023). Research indicates that its dysregulation may lead to abnormal cleavage of the APP (Qin et al., 2003; Guan and Wang, 2019), even creating a vicious cycle (Quadros et al., 2003). Exercise can inhibit the COX-2 pro-inflammatory pathway. Studies indicate that exercise significantly reduces COX-2 activity, potentially exerting therapeutic effects on vestibular migraine by suppressing COX-2-mediated inflammation (Lee et al., 2015). Moreover, moderate-intensity exercise can also reduce levels of COX-2 and APP (Pahlavani, 2023). This provides a theoretical basis for the potential therapeutic effect of exercise on AD by inhibiting COX-2, thereby preventing abnormal cleavage and the level of the APP. It also offers a direction for future research. Investigating whether exercise can indeed inhibit COX-2 in AD models, effectively prevent abnormal cleavage of APP, and reduce pathological products such as Aβ plaques may become a key focus for future research.

### GAPDH

Glyceraldehyde-3-phosphate dehydrogenase (GAPDH) has long been recognized as a crucial enzyme in energy metabolism, generating ATP and pyruvate through anaerobic glycolysis in the cytoplasm (Nicholls et al., 2012). Recent studies have progressively elucidated the crucial role of GAPDH in non-metabolic processes. Whether it is promoting the antitumor immune activity of CD8 T cells (Wang X. et al., 2024), acting as a housekeeping gene with oncogenic effects across various cancers (Wang J. et al., 2023), or participating in the heme maturation of myoglobin and hemoglobin, these findings reveal its significant biological importance beyond metabolic functions. In the pathogenesis of AD, GAPDH also plays a pivotal role (El Kadmiri et al., 2014). Research indicates that GAPDH can form high-affinity interactions with APP, which may be responsible for impaired glycolytic function in AD and could ultimately lead to cell death (El Kadmiri et al., 2014; Ahmad et al., 2023). However, limited research indicates that exercise can prevent the upregulation of GAPDH (Zhao et al., 2012). However, whether this mechanism can reduce the interaction between GAPDH and APP, thereby delaying the progression of AD, remains to be further investigated. Metabolism is a complex, multi-pathway biological process. To determine whether exercise can regulate APP by inhibiting GAPDH upregulation, it is necessary to observe whether GAPDH-APP interactions are weakened post-exercise, assess downstream GAPDH responses, and examine whether related metabolic processes like glycolysis regain function. Concurrently, monitoring changes in APP processing and assembly into Aβ is also essential.

### BACE-1

β-amyloid precursor protein-cleaving enzyme 1 (BACE-1) is a novel type I transmembrane aspartic protease essential for the generation of Aβ peptides in AD (Venugopal et al., 2008; Singh et al., 2022a). BACE-1 is widely distributed throughout the central nervous system (CNS) and exhibits all functional characteristics of a β-secretase, acting as the key enzyme initiating Aβ formation (Luo et al., 2016) Increased neuronal BACE-1 activity is believed to promote the production of Aβ and the formation of amyloid plaques in the brains of AD patients (Singh et al., 2022b). BACE-1 cleaves the APP into soluble APP and the membrane-bound C-terminal fragment C99, a rate-limiting step. C99 is further cleaved by γ-secretase to produce Aβ (Gehlot et al., 2022). The beneficial effects of exercise on AD likely also stem from its regulation of BACE-1. Studies indicate that treadmill exercise (TE) prevents PS2 mutation-induced memory deficits and reduces Aβ-42 deposition by inhibiting β-secretase and its product C-99 in the cortex and/or hippocampus of aged PS2 mutant mice (Kang et al., 2013). This partially explains exercise’s inhibitory effect on BACE-1, indirectly suggesting that exercise may effectively regulate the processing changes of APP. However, current research evidence remains insufficient and requires further clarification and validation. For instance, experimental investigation into whether the non-amyloidogenic pathway is activated and experiments such as measuring APP content and APP gene transcription expression levels would enhance our understanding of the underlying mechanisms.

### Cathepsins B, D, and E

Cathepsins are a class of proteases found within cells of various animal tissues and constitute major members of the cysteine protease family. Approximately 11 cathepsin isoforms are primarily present in the human body, with cathepsins B, D, E, and others having been demonstrated to be closely associated with AD pathogenesis. Cathepsins B, D, and E are all capable of metabolically degrading Aβ peptides. Among these, cathepsin D has been observed to cleave APP100-FLAG near the C-terminal end of the Aβ peptide. Meanwhile, cathepsin B exhibits high carboxypeptidase activity under low pH conditions. Therefore, the possibility that cathepsins D and B participate in the amyloid processing of APP cannot be ruled out (Mackay et al., 1997). Numerous studies have demonstrated that exercise can increase peripheral tissue protease B levels and improve cognitive function (Gökçe and Gün, 2023). However, whether cathepsins are involved in the process by which exercise improves cognitive function requires further research to elucidate.

### ERK/CREB

Extracellular signal-regulated kinase (ERK) is a member of the mitogen-activated protein kinase (MAPK) family. CAMP response element-binding protein (CREB), which selectively binds to cAMP response elements (CRE) and regulates gene transcription in various cells, including dopaminergic neurons (Zhen et al., 2023). The ERK/CREB pathway has been increasingly elucidated to play a crucial role in the nervous system. Its function in mediating neurotrophic factors and exerting neuroprotective effects has been widely recognized (Grimes et al., 2015). ERK/CREB signaling regulates synaptic protein expression and learning and memory functions (Peng et al., 2010; Wang J. et al., 2023). The progression of AD is often accompanied by memory decline. A study on treadmill exercise has also demonstrated that it can effectively activate the ERK/CREB pathway (Seo et al., 2021). Although this study is limited to investigating signaling pathways and does not further clarify whether exercise can improve memory by activating the ERK/CREB pathway, it strongly suggests that exercise may enhance memory through this pathway. ERK is closely associated with APP, which was discovered long ago to potentially regulate ERK signaling (Venezia et al., 2006). Subsequent studies have further revealed that Ginsenoside Rg1 promotes non-amyloidgenic cleavage of APP via estrogen receptor signaling to MAPK/ERK (Shi et al., 2012). Additionally, studies have revealed that presenilin not only influences the processing of APP and the generation of Abeta, but also modulates the activity of the ERK through a protein kinase C alpha-dependent mechanism (Dehvari et al., 2008). This further adds to the complexity of the relationship between ERK and APP. Meanwhile, CREB has been found to exert transcriptional regulation over PEN-2, a key component of the γ-secretase complex (Wang et al., 2006). Not only ERK protein, but also the activation of the ERK/CREB pathway has been elucidated in studies as a crucial intermediate step in the non-amyloid cleavage and metabolism of APP (Guo et al., 2017). Therefore, exercise may exert potential therapeutic effects on AD by promoting the non-amyloid cleavage and processing of APP through effective activation of the ERK/CREB pathway. This warrants further investigation and validation by researchers in their studies.

## APP assembly process and Aβ changes in exercise injury related diseases

### Fracture

Fractures rank among the most common sports injuries. An intriguing question arises: Could sports-related fractures alter the assembly of APP, leading to abnormalities in learning, memory, and cognitive function and potentially even AD? This curiosity drives researchers and may concern fracture patients. While current research remains limited, existing studies offer some preliminary insights into this question, that it may affect cognitive function (Guo et al., 2014). Clinical studies directly reveal that among hip fracture patients without dementia, 88.6% of cognitively normal individuals exhibit partial or complete abnormalities in indicators such as the Aβ42/40 ratio, p-tau, and t-tau (Oh et al., 2018). Moreover, more in-depth research has focused on APP, revealing a potential shared molecular pathway in disease progression between femoral neck fractures, osteoporosis, and AD by investigating the close relationship between APP genes and bone remodeling genes such as TRAP and RANKL (Stapledon et al., 2021). Of course, current research has not revealed whether changes in the APP processing and assembly process occur during the injury itself or during the subsequent repair process. This research question presents numerous challenges awaiting breakthrough solutions. Among these, how to eliminate other influencing factors and determine whether fracture injury is one of the primary factors causing changes in APP assembly is a key issue requiring resolution. Additionally, the process of APP assembly changes as injury occurs and recovery progresses is a fascinating topic equally worthy of in-depth investigation.

### Osteoporosis

Osteoporosis is a common disease that contributes to sports injuries. Previously, we often believed that this condition primarily affected the elderly or postmenopausal women (Johnston and Dagar, 2020; Walker and Shane, 2023). However, with the continuous evolution of lifestyle habits, osteoporosis has shown a trend toward younger onset, and the management of younger patients has become a difficult challenge to address (Herath et al., 2022; Ishimoto and Shah, 2024). We note that previous studies have found significantly elevated levels of Aβ in bone tissue affected by osteoporosis (Downey et al., 2017; Duan et al., 2023). Osteoporosis may also affect cognitive function (Zhao et al., 2023). This establishes a link between osteoporosis and Aβ, thereby paving the way for further investigation into the potential relationship between osteoporosis and AD (Wang H. S. et al., 2024; Nasme et al., 2025). However, this potential link remains uncertain unless elevated levels of Aβ are simultaneously detected in brain tissue from individuals with osteoporosis. This finding also suggests that osteoporosis may influence Aβ levels by affecting the assembly process of APP. While current research has not elucidated the underlying mechanisms, it points the way for future investigations.

### Muscle injury

Muscle injuries rank among the most common conditions resulting from sports-related trauma. This category encompasses numerous distinct types of injuries. Among them, muscle strains are relatively more prevalent and generally easier to recover from. This group also includes more severe conditions like rhabdomyolysis, which can cause multi-organ involvement. Many such cases involve irreversible damage and tend to have a relatively slow recovery process (Guerrero-Hue et al., 2023; Nathan, 2023). Research indicates that muscle strains may lead to cognitive impairment (Guéniot et al., 2020). The connection between muscle mass and Aβ is strong. From a clinical research perspective, greater thigh muscle mass is associated with a lower risk of Aβ positivity in women (Kang et al., 2022). From a basic research perspective, intracellular accumulation of Aβ leads to Ca^2+^ dysregulation in skeletal muscle (Lopez and Shtifman, 2010). Moderate exercise can aid skeletal muscle energy metabolism, but inappropriate exercise may lead to certain traumatic injuries (Baumert et al., 2016; Yook and Cho, 2017). During this process, certain metabolic pathways may be disrupted. Recent research has focused intensely on the close association between exercise-induced myopathy and Aβ deposition. Findings suggest a potential strong link between severe exercise-induced myopathy and tissue infiltration by amyloid deposits in skeletal muscle (Appelman et al., 2024). APP, as the precursor of Aβ, is located at the base of the postsynaptic folds of the neuromuscular junction and is regulated by the neurotrophic factor neurotrophic factor-1 (NRG1). NRG1 causes a reduction in APP mRNA levels while simultaneously decreasing steady-state protein levels (Rosen et al., 2003). In skeletal muscle, the absence of NRG1 leads to the loss of neuromuscular synapses (Liu et al., 2019). Interestingly, NRG1 expression increases within the first 48 h after muscle injury, aiding in the remodeling and recovery of both presynaptic and postsynaptic regions (Pimentel Neto et al., 2025). This implies that during the initial phase of muscle injury, APP expression is likely influenced by increased NRG1 expression, resulting in a decrease in its levels. Whether APP levels rebound after 48 hours and whether Aβ deposition increases remains to be further investigated and discovered. For severe exercise-related muscle injury diseases such as rhabdomyolysis, the connection between these conditions and Aβ deposition represents an intriguing research direction; however, currently reported studies in this area remain limited.

**FIGURE 3:**
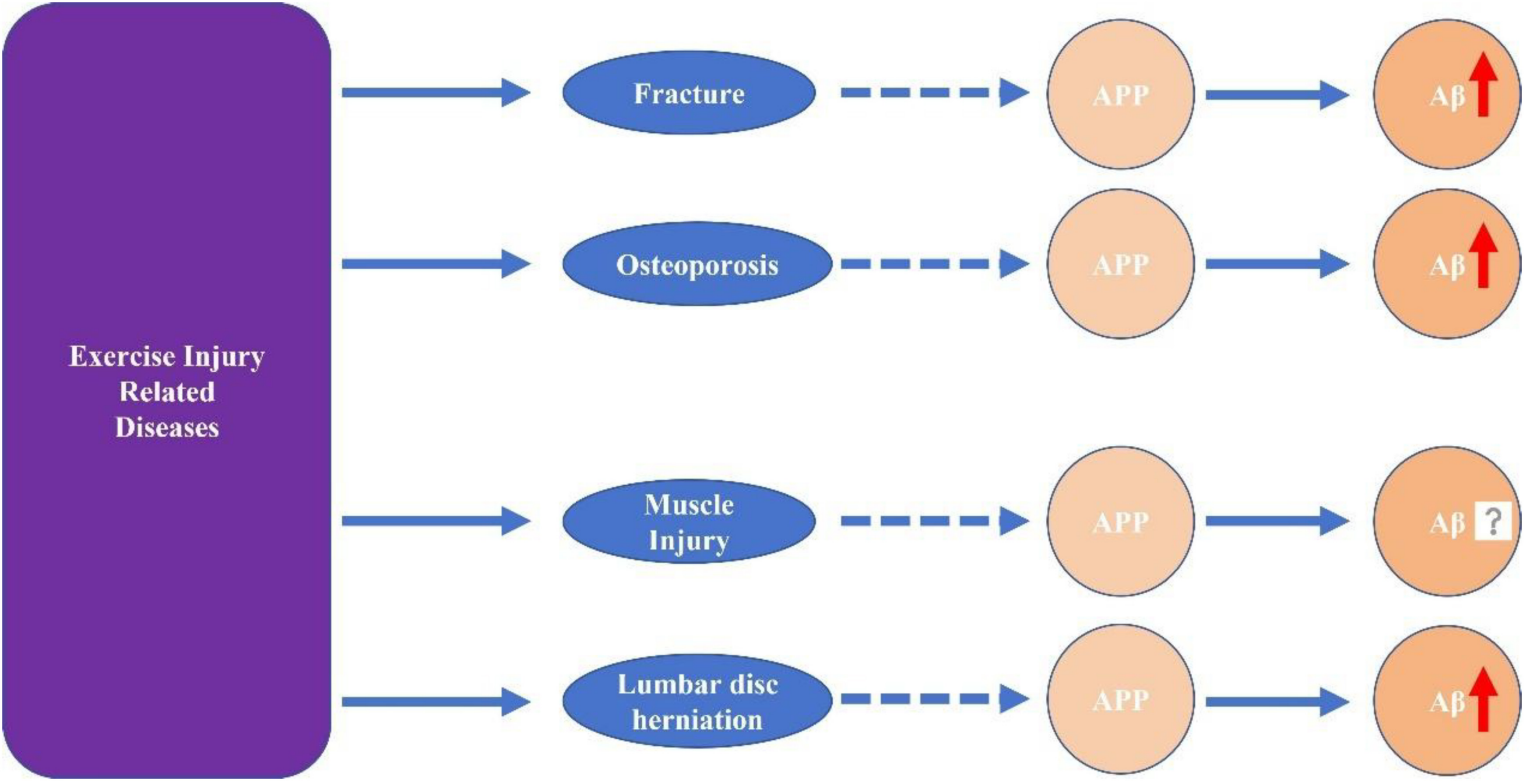
The potential link between exercise-related injuries and alterations in β-amyloid precursor protein (APP) assembly and β-amyloid (Aβ) pathology.

The variety of diseases caused by exercise injuries is extensive, making it difficult to list them all in detail. Among these, some diseases may exhibit alterations in the APP processing pathway during their progression. Currently, most research on these diseases lacks findings that directly clarify the relationship between APP processing, Aβ generation and deposition, and disease progression. However, we can still discern a close connection between these diseases and cognitive impairment from a few studies, such as those on lumbar disk herniation (Luo et al., 2025). This underscores the value and significance of investigating mechanisms related to APP and Aβ (Figure 3).

## Conclusion

Exercise is an integral part of everyone’s daily life. Currently, there is a lack of in-depth research evidence on whether the exercise process affects the processing and assembly of APP. However, this does not diminish the importance of studying this issue. It will help us gain a more comprehensive and profound understanding of exercise’s role in improving cognition and its impact on altering the progression of AD. Physical activity inevitably carries the risk of sports injuries. Regardless of their severity, such injuries may disrupt the processing and assembly of APP, potentially affecting cognition and even accelerating disease progression. Research in this direction holds equally significant value and importance. Clarifying whether sports injuries can impact cognition by affecting APP processing and assembly will help us avoid adverse outcomes.
